# The effect of rehabilitation on quality of life in female breast cancer survivors in Iran

**DOI:** 10.4103/0971-5851.76190

**Published:** 2010

**Authors:** M. Poorkiani, A. Abbaszadeh, M. Hazrati, P. Jafari, M. Sadeghi, M. Mohammadianpanah

**Affiliations:** *Department of Nursing, College of Nursing and Midwifery, Kerman University of Medical Sciences, Kerman, Iran*; 1 *Department of Nursing, College of Nursing and Midwifery, Shiraz University of Medical Sciences, Shiraz, Iran*; 2 *Department of Biostatistics, Shiraz University of Medical Sciences, Shiraz, Iran*; 3 *Department of Physiotherapy, Kerman University of Medical Sciences, Kerman, Iran*; 4 *Department of Radiotherapy, Nemazi Hospital, Shiraz University of Medical Sciences, Shiraz, Iran*

**Keywords:** *Breast cancer*, *quality of life*, *rehabilitation*

## Abstract

**Background:**

The purpose of this study was to compare the quality of life (Qol) of female breast cancer survivors who received rehabilitation intervention beside medical care and survivors who received medical care alone.

**Materials and Methods:**

Fifty-seven female breast cancer survivors were assigned to usual medical care (control group) or to usual medical care plus rehabilitation intervention (experimental group). Qol of all patients was assessed before, 1 week and 3 months after intervention. The intervention consisted of physiotherapy, education and individual counseling. The authors used the European Organization for Research and Treatment of Cancer core questionnaire and breast module (EORTC QLQ-C30/BR23) for the assessment of Qol.

**Results:**

Patients who received rehabilitation had significantly better Qol. Overall, mean of Qol scores improved gradually in experimental group from before to 1 week and 3 months after intervention. In contrast, minimal change was observed between pre/post and follow-up measures for control group.

**Conclusion:**

Rehabilitation after breast cancer treatment has the potential for physical, psychological and overall Qol benefits.

## INTRODUCTION

Breast cancer is one of the most common malignancies affecting women worldwide.[1] Because of modern treatment options, more and more women are being cured of their malignancies.[2] Although mortality rate and the number of people dying from the breast cancer have declined[3] and survival rate has increased due to early detection, with advanced technology and effectiveness of current treatment plans,[4] however, a lot of patients suffer from treatment side effects. In the immediate postoperative period, many problems might occur, such as the limitation of shoulder motion, edematous arm, numbness of chest wall and arm, and depression.[5] In addition, most patients might feel disabled due to the loss of their breast, distorted body image or self-concept, change in relationships with their husband and families, fair of recurrence on the disease or death.[6] These symptoms might decrease during treatment but they still can be significant factors resulting in discomforts in daily living and decreasing the quality of life (Qol).

Health-related Qol is a multidimensional term, which is generally used as a health description. It consists of different domains such as physical and social functioning and psychological well-being.[7] Although investigating Qol as an outcome is challenging because it is multidimensional, involves complex, interrelating factors and is subjective,[8] over the years, most treatment options for breast cancer (palliative or adjuvant therapies) have been evaluated for their impact on Qol.[9]

Colman (1984) hypothesized that Qol in cancer patients represents the difference between the hopes and expectation of an individual and the actual experience of their present situation. Perhaps it is the role of rehabilitation to lessen the gap between these two realities.[10] Rehabilitation interventions can help maximize the functional status of individuals with breast cancer and reduce the morbidity associated with the disease and its treatment. It can also address the psychosocial and vocational problem associated with breast cancer and lead to improvements in well-being and Qol.[11] If the patients receive suitable rehabilitation support services, they will be able to fulfill social and occupational roles while undergoing active cancer treatment.[12] Therefore, attention to the functional problems of breast cancer patients is relevant at any point in the diagnostic and therapeutic continuum and rehabilitation interventions are appropriate for all of these individuals who are living with cancer.[11]

Nurses play a major role in the rehabilitation of the patients with cancer. They frequently provide case-management and patient education services and facilitate support for these groups.[13]

Most information regarding the effect of rehabilitation on Qol of breast cancer patients originate from research in western countries. In Iran, rehabilitation program is not a part of usual treatment of breast cancer patients and a lot of physicians believe that rehabilitation cannot improve side effects of breast cancer and treatment. So, due to lack of these studies in Iranian women, it is difficult to know whether similar conclusions can be drawn across cultural boundaries. The aim of this study was to determine whether Iranian patients who received post mastectomy rehabilitation program showed an improved Qol compared to a group of patients who received medical care only.

## MATERIALS AND METHODS

### Participants

This study was conducted as a clinical trial. Patients (*n*=66) were female breast cancer survivors of Nemazi Hospital in Shiraz, Iran. Patients’ criteria included the following: those who had undergone modified radical mastectomy surgery for one time, had finished primary treatment (surgery, chemotherapy and radiotherapy) at least 6 months before enrolling in the study, and were receiving hormone therapy. None of the patients had any kind of illness or physical problem that restricted rehabilitation programs.

### Procedure

Qol was assessed before the beginning of the rehabilitation program, 1 week and 3 months after the program. Patients’ Qol in both experimental and control groups were compared with each other.

### Sample size

According to same method of study size was determined as 27 people in each group. In order to prevent sample attrition of 20%, the number of the samples was fixed as 33 patients in each group. Then, eligible patients were randomly assigned to the control and the experimental groups. During the intervention, five people from experimental group and three from control group were omitted due to metastasis and restart of the treatments. Also, one patient from experimental group dropped out of the study at follow-up stage due to unknown reasons.

### Interventions

After the first data gathering, the experimental group underwent rehabilitation programs such as physiotherapy, education and consultation beside medical care. Physiotherapy included electrotherapy, exercises and massage therapy done during 10–30 sessions three times per week in order to reduce pain, arm lymphedema and to increase the shoulder range of motion. Education was given individually and face to face according to patients’ educational needs during two to four sessions of duration 45–90 minutes.

At the end, instructed materials were given to patients in the form of instructional pamphlets. A nurse who was an expert in psychoanalysis held one to three consultation sessions of 30–60 minutes duration, individually. The whole intervention lasted for 2 months and in this period no particular rehabilitation program was done on the control group. One week and 3 months after the interventions, the patients’ Qol in both experimental and control groups was reexamined and the results of each examination were compared with each other. After the final stage of data collection, the required education and educational pamphlets were presented to the patients in the control group and for the ethical issues to be observed, those patients who needed counseling and physiotherapy were referred to the relevant experts.

Two general Qol questionnaires related to cancerous patients, European Organization for Research and Treatment of Cancer core questionnaire (EORTC QLQ-C30) and a specific questionnaire related to breast cancer patients’ Qol, EORTC QLQ-BR23, were used in order to measure patients’ Qol. These questionnaires include symptom and functional scales and each of these scales contains a set of questions. The acquired scores of each scale are spread in the 0–100 domain. A higher score in the functional scales indicates a better function, and in the symptom scales, it indicates a more intensive symptom. Both the questionnaires were of the standard type and had been used in various studies. The reliability and the validity of the Persian version of the questionnaires had been examined by Montazeri *et al*. (1999–2000) in Iran and the Persian version of these questionnaires had been introduced by European Society of Cancer Research and Treatment as a reliable and valid instrument.[14,15] Data were analyzed by statistical tests such as Chi-square, Fisher test, Paired *t*-test, independent *t*-test and repeated-measure analysis of variance (ANOVA). A *P* value less than or equal to 0.05 was considered as statistically significant.

## RESULTS

A total of 57 patients in the form of experimental and control groups participated in this research. The average age of the patients was 40.7 years in the experimental group and 36.7 years in the control group. Most of the cases were married (88.9 in experimental group and 83.3 in the control group) with under diploma level of education. Chi-square test and Fisher test showed no statistically significant difference between the two groups in terms of age, marital status and education and time of surgery.

The analysis of the results before the intervention showed that both groups were homogenous in terms of Qol, and from 15 symptoms and functional scales under the study, only two fields of physical (*P*=0.000) and emotional function (*P*=0.020) showed a statistically significant difference between experimental and control groups. A comparison of the results of Qol examination in relation to breast cancer indicates that before the beginning of the intervention, there was a statistically significant decrease in the body image (*P*=0.000), future perspective (*P*=0.001) and arm symptoms (*P*=0.000), and statistically significant increase in the scale of sexual enjoyment (*P*=0.025) in the experimental group compared to the control group [Tables 1 and 2].

**Table 1:** Functional and symptom outcome (means, standard deviation) derived from EORTC QLQ-C30 in experimental and control groups, 1 week after intervention

	Experimental group Means±SD	Control group Means±SD	*P* value
Functional scales			
Global health	57.4±14.85	40.25±8.49	0.000
Physical functioning	65.18±45.9	65.33±12.79	0.987
Role functioning	78.39±15.88	64.44±19.44	0.005
Emotional functioning	60.49±18.71	45±19.27	0.003
Cognitive functioning	75.3±21.36	60.55±23.35	0.016
Social functioning	68.51±23.72	58.88±23.46	0.129
Symptom scales			
Fatigue	26.33±15.45	42.22±21.1	0.003
Nausea and vomiting	2.46±7.6	3.88±12.33	0.603
Pain	32.71±20.4	45±25.20	0.050
Dyspnea	8.64±14.88	17.77 ±25.88	0.113
Insomnia	22.22±18.49	26.66±30.82	0.518
Appetite loss	4.93±12.6	12.22±23.94	0.160
Constipation	4.93±15.2	10±21.7	0.317
Diarrhea	2.46±8.89	4.44±11.52	0.476
Financial difficulties	27.16±27.79	27.77±30.42	0.937

**Table 2:** Functional and symptom outcome (means, standard deviation) derived from EORTC QLQ-BR23 in experimental and control groups, 1 week after intervention

	Experimental group Means±SD	Control group Means±SD	*P* value
Functional scales			
Body image	45.98±16.4	48.05±17.18	0.645
Sexual functioning	48.61±18.98	61.33±14.20	0.011
Sexual enjoyment	47.22±16.78	60±21.51	0.025
Future perspective	54.32±18.82	47.77±22.63	0.243
Symptom scales			
Systemic therapy side effects	13.93±9.14	20.31±15.76	0.071
Breast symptoms	22.22±20.92	28.5±17.01	0.251
Arm symptoms	46.91±16.69	54.44±15.53	0.083
Upset by hair loss	23.80±25.19	33.33±35.63	0.566

One week after the rehabilitation process, the experimental group had an increase in all general Qol functional scales and had a decrease of symptoms scales compared to the control group. These changes had been statistically significant in functional scales of global health (0.000), role function (0.005), emotional function (0.003), cognitive function (0.016), symptom scales of fatigue (0.003), and pain (0.050). Results of breast cancer patients’ Qol in the fields of sexual function (0.011) and sexual enjoyment (0.025) were significant and experimental group’s function was worse than the control group [Tables 3 and 4].

**Table 3:** Functional and symptom outcome (means, standard deviation) derived from EORTC QLQ-C30 in experimental and control groups, 3 months after intervention

	Experimental group Means±SD	Control group Means±SD	*P* value
Functional scales			
Global health	69.13±12.4	43.05±43.05	0.000
Physical functioning	85.18±9.3	64.22±19.21	0.000
Role functioning	88.88±14.61	63.33±21.62	0.000
Emotional functioning	81.48±14.12	38.05±32.73	0.000
Cognitive functioning	87.65±15.04	57.77±29.27	0.000
Social functioning	85.80±15.81	55±23.63	0.000
Symptom scales			
Fatigue	9.05±10.6g	48.14±23.58	0.000
Nausea and vomiting	0.00±0.00	5±15.87	0.108
Pain	11.72±14.48	47.77±27.93	0.000
Dyspnea	7.4±14.12	53.33±25.67	0.000
Insomnia	13.58±19.7	25.55±29.92	0.081
Appetite loss	0.00±0.00	11.11±23.7	0.018
Constipation	0.00±0.00	10±23.40	0.031
Diarrhea	1.23±6.41	30±35.39	0.180
Financial difficulties	28.39±38.89	30±35.39	0.871

**Table 4:** Functional and symptom outcome (means, standard deviation) derived from EORTC QLQ-BR23 in experimental and control groups, 3 months after intervention

	Experimental group Means±SD	Control group Means±SD	*P* value
Functional scales			
Body image	71.6±10.9	43.33±35.31	0.000
Sexual functioning	40.97±17.01	60±19.24	0.001
Sexual enjoyment	40.57±14.05	56.06±23.87	0.011
Future perspective	69.13±18.31	38.88±32.85	0.000
Symptom scales			
Systemic therapy side effects	8.81±7.07	21.11±16.19	0.001
Breast symptoms	11.41±13.3	30.27±21.71	0.000
Arm symptoms	28.8±13.03	60±22.33	0.000
Upset by hair loss	22.22±19.24	45.83±35.35	0.311

Three months after the intervention, the experimental group had a significant functional increase in all functional scales of general Qol compared to control group. In symptom scales also, all the symptoms had been reduced compared to control group. This decrease had been significant in scales of fatigue (0.000), dyspnea (0.000), pain (0.000), anorexia (0.018), and constipation (0.031). Therefore, in sum, the two groups had significant statistical difference in 11 domains. Also, the experimental group had shown a significant recovery. In terms of Qol related to breast cancer, experimental group had a significant recovery in scales of body image (0.000), future perspective (0.000), systemic therapy side effects (0.001), breast symptoms (0.000), and arm symptoms (0.000). The experimental group had a decrease in scales of sexual function (0.001) and sexual enjoyment (0.011) compared to the control group.

## DISCUSSION

Breast cancer is one of the most widespread cancers in women. Due to the disease process and treatment-related adverse effects, the patients’ Qol is affected. Therefore, the provision of appropriate treatment and care programs for controlling the disease and improving the patients’ Qol is a fundamental factor. In this study, we investigated the role of rehabilitation processes such as education, consultation and physiotherapy on patients’ Qol improvement.

Generally, the change in the trend of general Qol and Qol in relation to breast cancer in the time period before and 3 months after intervention indicates that the rehabilitation process had been effective on patients’ Qol in the experimental group. No significant change occurred in the patients’ Qol in the control group in the third period and it can be said that the condition of the patients in this group was stable, and in some fields such as dyspnea, it became worse.

Before rehabilitation intervention, the experimental group had a significant decrease in the fields of physical, emotional function and general Qol compared to the control group; however, 1 week after the intervention, this group had a significant improvement in six fields, and 3 months later, there was improvement in 11 fields. Therefore, it can be concluded that the intervention had a positive effect on the experimental group and patients’ Qol in the experimental group improved significantly.

The trend in improved results in the experimental group indicates that time plays a key role in the assessment of the effect of rehabilitation intervention on the Qol. The results of Strauss’ study (2006) showed that 6 months after the interventions, general Qol in breast cancer patients in functional scales had increased and the symptoms scales and pain in particular had decreased.[16] The results of Parks’ study (2006) in Korea also indicate a significant increase of Qol of experimental group, 10 weeks after the rehabilitation, while there was no significant change in Qol of the control group in this time period. In addition, 22 weeks after the rehabilitation, the results showed a significant increase in the Qol of the experimental group and a significant decrease in the control group.[17]

The analysis of the results, 1 week to 3 months after the treatment, indicated a decrease in the sexual function and sexual enjoyment of the experimental group compared to the control group. With regard to the fact that the control group had no significant changes in these fields before, 1 week and 3 months after the intervention, the significant difference between the two groups had been due to the functional decrease of the experimental group. However, many scholars believe that patients’ answers to the sexual questions are not so reliable and often they cannot judge based on those responses.[18] Perhaps we can justify the results with regard to the fact that all the scales of Qol were subjective and exclusively related to the patient. In the domains of sexual functions, patients’ spouses’ satisfaction with sexual function also plays a direct role in the decrease or increase of the function. Probably, patient’s recovery and her Qol improvement increase her sexual appeal, but this sense of sexual need is associated with sexual partner’s unwillingness and this inappropriate response produces the reverse results. Therefore, we can reinforce the physical, emotional, and affective power of the patient by appropriate sexual consultation, requiring the patients to participate in these programs and providing the patient and her partner with more time to adapt themselves to these issues.

It is also consistent with a report by Hazard (2005) that in the period of 1–3 months after the rehabilitation, no statistically significant change was observed in the sexual function of the experimental group.[19] Heravi krimway (2006) studied the effect of group counseling program on the sexual health of breast cancer survivors.[20] In this study, group counseling increased patients’ sexual function. The contradictory result of the studies indicates the need for more studies and researches to be conducted in this aspect.

The results of Fors *et al*. (2010), in a systematic review addressing the efficacy of psychosocial interventions, are different and summarizing the results from studies is difficult. Thus, more psychosocial researches are clearly necessary.[21]

In general, rehabilitation effect appeared in a shorter period on the functional scales of general Qol in the experimental group, so the positive effect of rehabilitation had been clear 1 week after the end of the intervention and this recovery process continued at the same rate, 3 months after the intervention. The negative effects of the breast cancer and its treatment on the Qol were higher for the Qol related to breast cancer and the desired response took more time to appear. Therefore, in order to analyze Qol of the breast cancer survivors, general Qol examination is not sufficient and examination of Qol in relation to breast cancer seems inevitable.

## CONCLUSION

The Qol of breast cancer survivors who had attended rehabilitation programs such as physiotherapy, education and counseling had been much better than those who had not attended these programs. Therefore, more attention should be paid to rehabilitation programs by holding consultation session for the patients and continuing nursing services for these patients. Therefore, patients’ release from the hospital should not be accounted as the end of the nursing care.
